# Supplementary material for: Subfunctionalization of tbx2 paralogues during photoreceptor cell specification in zebrafish
Source: bioRxiv. 2026 Aug 11:2026.07.01.735836. Preprint. [Version 3] doi: 10.64898/2026.07.01.735836 (PMC13640496; doi:10.64898/2026.07.01.735836)
Supplement: Supplementary file 1 [file media-1.pdf]

qPCR Gene	Primer Sequence (5'→3')
Lws1	TACCTGGCCATCCATGCTG
Lws1	GGCGAAGAACGTGTAAGGAC
Lws2	ATGCCTGCCTACTTTGCC
Lws2	CCAGTTCTTCCCTCTTGTTC
Rh2-2	GCGTGGGTAGATTAGTTGTG
Rh2-2	GGCTTATGCTCAGATTTAGTGG
Rh2-3	AGGAAACAAAAACAGCATTG
Rh2-3	ATACAGTATAAATGACAGCCTTC
Sws1	CGAGAGATATGTGGTCATCTG
Sws1	TGTATCTGCTCCATCCAAAG
Sws2	GGAGGAATGGTGAGTTTGTG
Sws2	GGTCTTGAAGGTAAAGTTCC
Rho	TCCGAGACCACACAGCG
Rho	CTGCTTGTTCATGAGATG

Supplementary Table 3: Primer sequences used for qPCR.
