# Supplementary material for: Subfunctionalization of tbx2 paralogues during photoreceptor cell specification in zebrafish
Source: bioRxiv. 2026 Aug 11:2026.07.01.735836. Preprint. [Version 3] doi: 10.64898/2026.07.01.735836 (PMC13640496; doi:10.64898/2026.07.01.735836)
Supplement: Supplementary file 2 [file media-2.pdf]

Gene	Primer Sequence (5'→3')
tbx2a exon 1	CATCACCCTATGCAGCATTC
tbx2a exon 1	CTTTCCCATGTCAGCTTTCC
tbx2a exon 2	GGGCTATCTGGGTGAATGTA
tbx2a exon 2	TGGTTATAACGGGAAAGGTATAGT
thrβ2 exon 1	TCCACTGCATCTGAGAGAAATC
thrβ2 exon 1	GTTCATTGTGTGCGGGATTG
nrl tss	TCAGCGCCGTCATGATTT
nrl tss	CGACGGTACGTCTGAGTTTATT
nrl exon 2	GAGATGACCGGTTCTCCATTC
nrl exon 2	GTGTCAGATCATACCTGTGAAGT
nrl exon 3	GAGTGGGAACCCTGCTTTAAT
nrl exon 3	TGTGTGAACGAGTGTGTATGG
six7 exon 1	GTTGGTTGTCCGTTACTCAC
six7 exon 1	ACTTCTCTACCGGGCCGAG

Supplementary Table 2: DNA primer sequences used for screening lesions across different loci.
