# Supplementary material for: Subfunctionalization of tbx2 paralogues during photoreceptor cell specification in zebrafish
Source: bioRxiv. 2026 Aug 11:2026.07.01.735836. Preprint. [Version 3] doi: 10.64898/2026.07.01.735836 (PMC13640496; doi:10.64898/2026.07.01.735836)
Supplement: Supplementary file 3 [file media-3.pdf]

Gene Target	gRNA Sequence (5'→3')
tbx2a exon 1	UUUCAAGGGUCUCGAGCCAGGUUUUAGAGCUAUGCU
tbx2a exon 2	GUUUCCUCCGUUCAAGGUGCGUUUUAGAGCUAUGCU
thrβ2 exon 1	CCAUCCUCCAAGCAUGGAGCGUUUUAGAGCUAUGCU
thrβ2 exon 1	CCACUGAACGGGCACCCAUAGUUUUAGAGCUAUGCU
nrl tss	GGGCCACUGUUACUGACAAUGUUUUAGAGCUAUGCU
nrl exon 2	UCUGGCCAACGUCAACCCGUGUUUUAGAGCUAUGCU
nrl exon 3	AUCUCCACCGGGUACAUCUCGUUUUAGAGCUAUGCU
six7 exon 1	ACGGUUGAGCAACUCUCCGGGUUUUAGAGCUAUGCU

Supplementary Table 1: gRNA sequences used for CRISPR/Cas9 targeting.
