# Supplementary material for: Subfunctionalization of tbx2 paralogues during photoreceptor cell specification in zebrafish
Source: bioRxiv. 2026 Aug 11:2026.07.01.735836. Preprint. [Version 3] doi: 10.64898/2026.07.01.735836 (PMC13640496; doi:10.64898/2026.07.01.735836)
Supplement: Supplementary file 4 [file media-4.pdf]

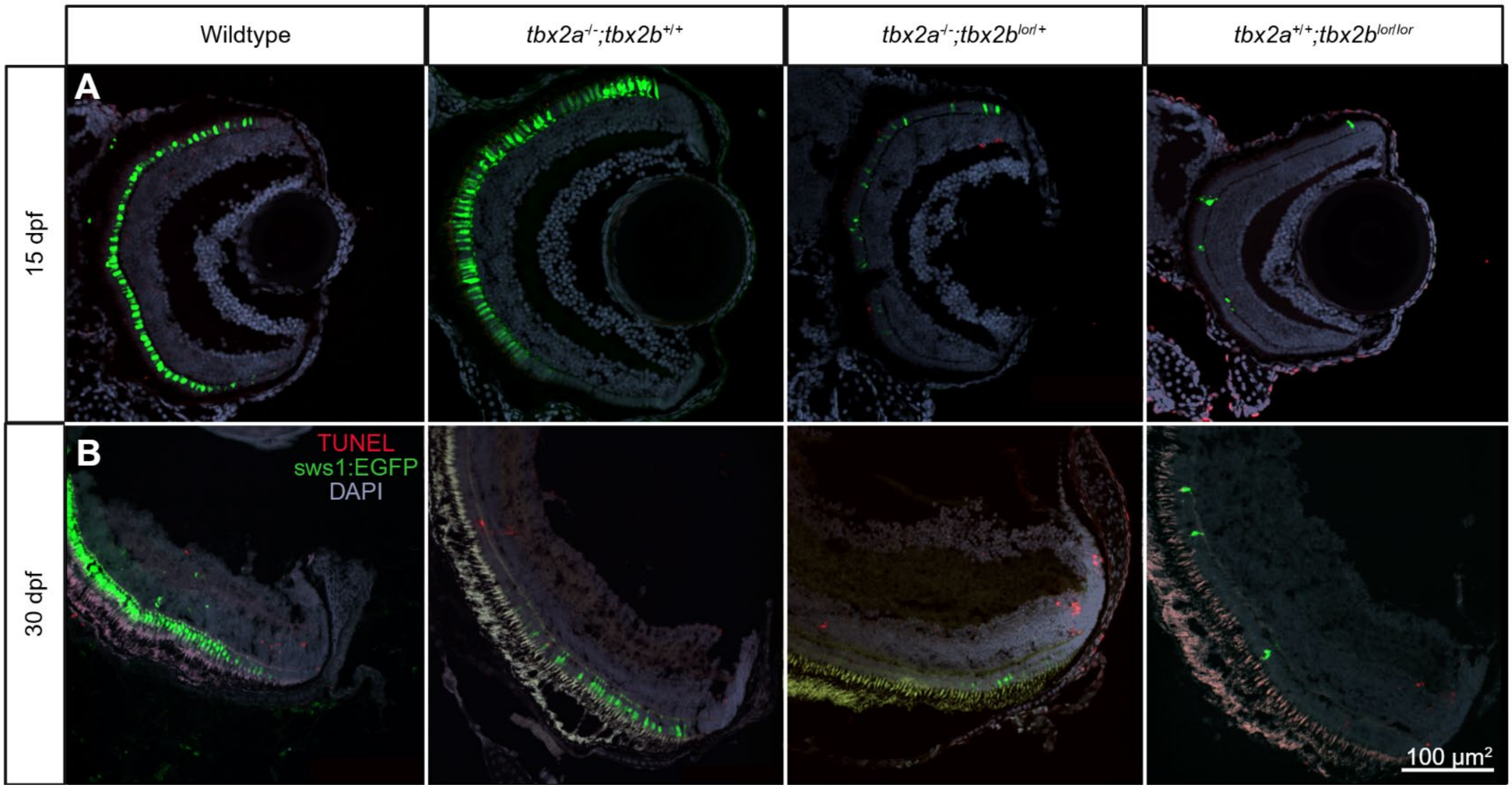

Supplementary Figure 3: Cell death was not observed at the boundary of sws1 cones. A-B) Immunolabeling for sws1EGFP (green) and TUNEL (Red) at 15 dpf (A) and 30 dpf (B) across genotypes. No difference was observed in the amount of TUNEL+ cells across genotypes.
