# Supplementary material for: Subfunctionalization of tbx2 paralogues during photoreceptor cell specification in zebrafish
Source: bioRxiv. 2026 Aug 11:2026.07.01.735836. Preprint. [Version 3] doi: 10.64898/2026.07.01.735836 (PMC13640496; doi:10.64898/2026.07.01.735836)
Supplement: Supplementary file 5 [file media-5.pdf]

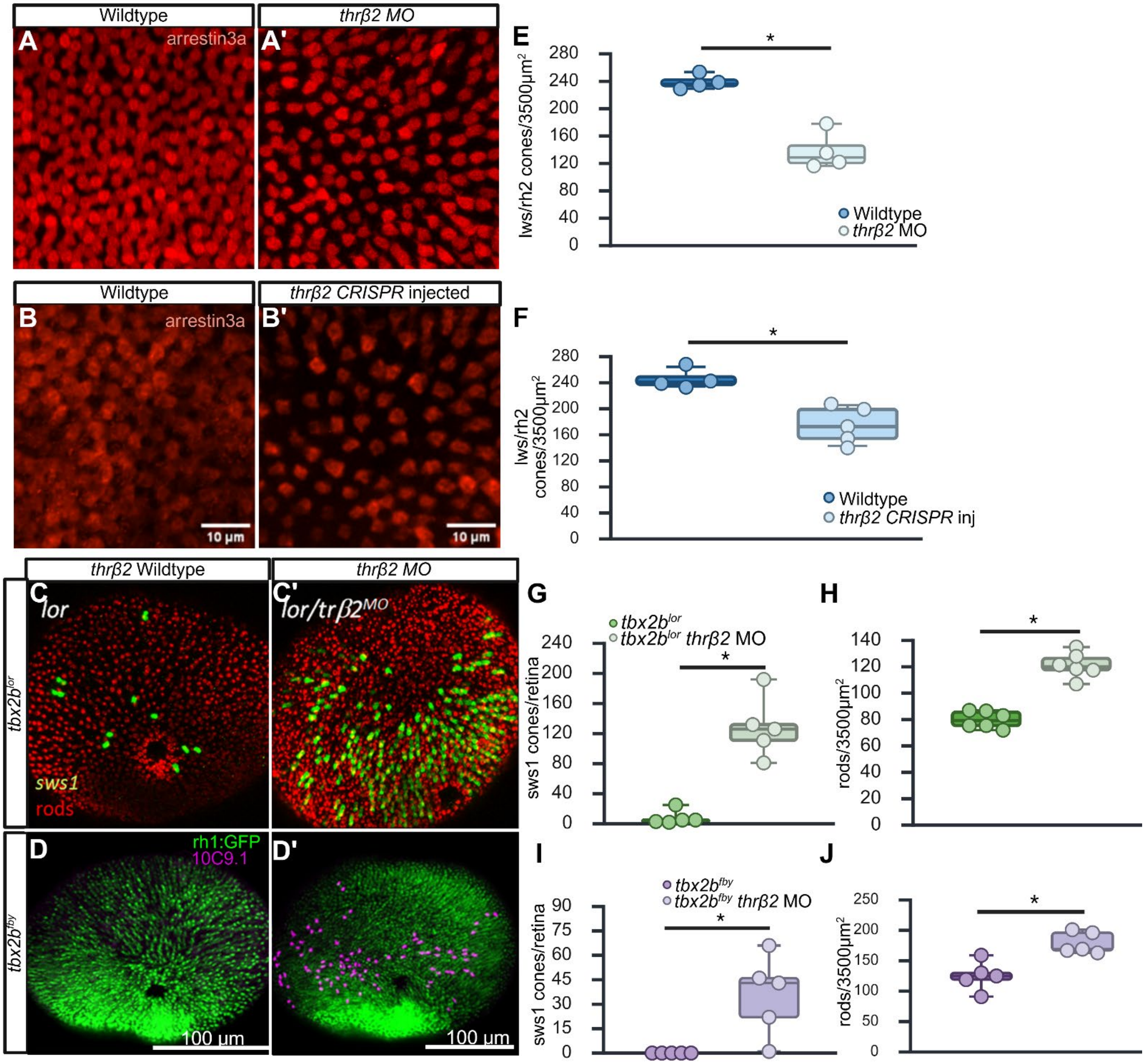

Supplementary Figure 2: *thrβ2* targeting using a morpholino or CRISPR/Cas9 leads to a significant reduction in rh2/lws cones and results in a significant increase in both sws1 cones and rods in *tbx2b* mutants. A-B) Wholemount immunolabeling for arrestin3a in 5 dpf wildtype embryos. Distribution of rh2/lws cones just dorsal the optic nerve in 5 dpf larvae. A'-B') Targeting of thrb2 using a morpholino or CRISPR/Cas9 resulted in a significant reduction in rh2/lws cones, attributed to the loss of lws cones. C) $tbx2b^{lor/lor}$ and D) $tbx2b^{fby/fby}$ animals have a reduced number of sws1 cones and a concomitant increase in rods. C') $tbx2b^{lor/lor}$ and D') $tbx2b^{fby/fby}$ animals injected with a morpholino specific to thrb2 resulted in a significant increase in both sws1 cones (purple) and rods (green). E-F) Quantification of lws/rh2 cones in morpholino (E): wildtype (n=4, 239+/-10.5), *thrβ2* MO (n=138+/-27.8) and *thrβ2* CRISPR (F) injected: wildtype (n=4, 245+/-15.5), thrb2 inj. (n=5, 175+/-28.5). G-J) Quantification of sws1 cones per retina (G: $tbx2b^{lor/lor}$ (n=5, 8+/- 9.6), $tbx2b^{lor/lor}$ thrb2 MO(n=5, 128+/-40.6))+ I: $tbx2b^{fby/fby}$ (n=5, 0), $tbx2b^{fby/fby}$ thrb2 MO (n=5, 36+/-24.8) and rods/3500 µm² (H: $tbx2b^{lor/lor}$ (n=6, 80+/-6.4), $tbx2b^{lor/lor}$ thrb2 MO (n=6, 121+/-9.6)+J: $tbx2b^{fby/fby}$ (n=5, 125+/-24.3), $tbx2b^{fby/fby}$ *thrb2* MO (n=5, 179+/-17.8) in morpholino injected animals. Student's unpaired t-test $p<0.05$.
