# Supplementary material for: Subfunctionalization of tbx2 paralogues during photoreceptor cell specification in zebrafish
Source: bioRxiv. 2026 Aug 11:2026.07.01.735836. Preprint. [Version 3] doi: 10.64898/2026.07.01.735836 (PMC13640496; doi:10.64898/2026.07.01.735836)
Supplement: Supplementary file 6 [file media-6.pdf]

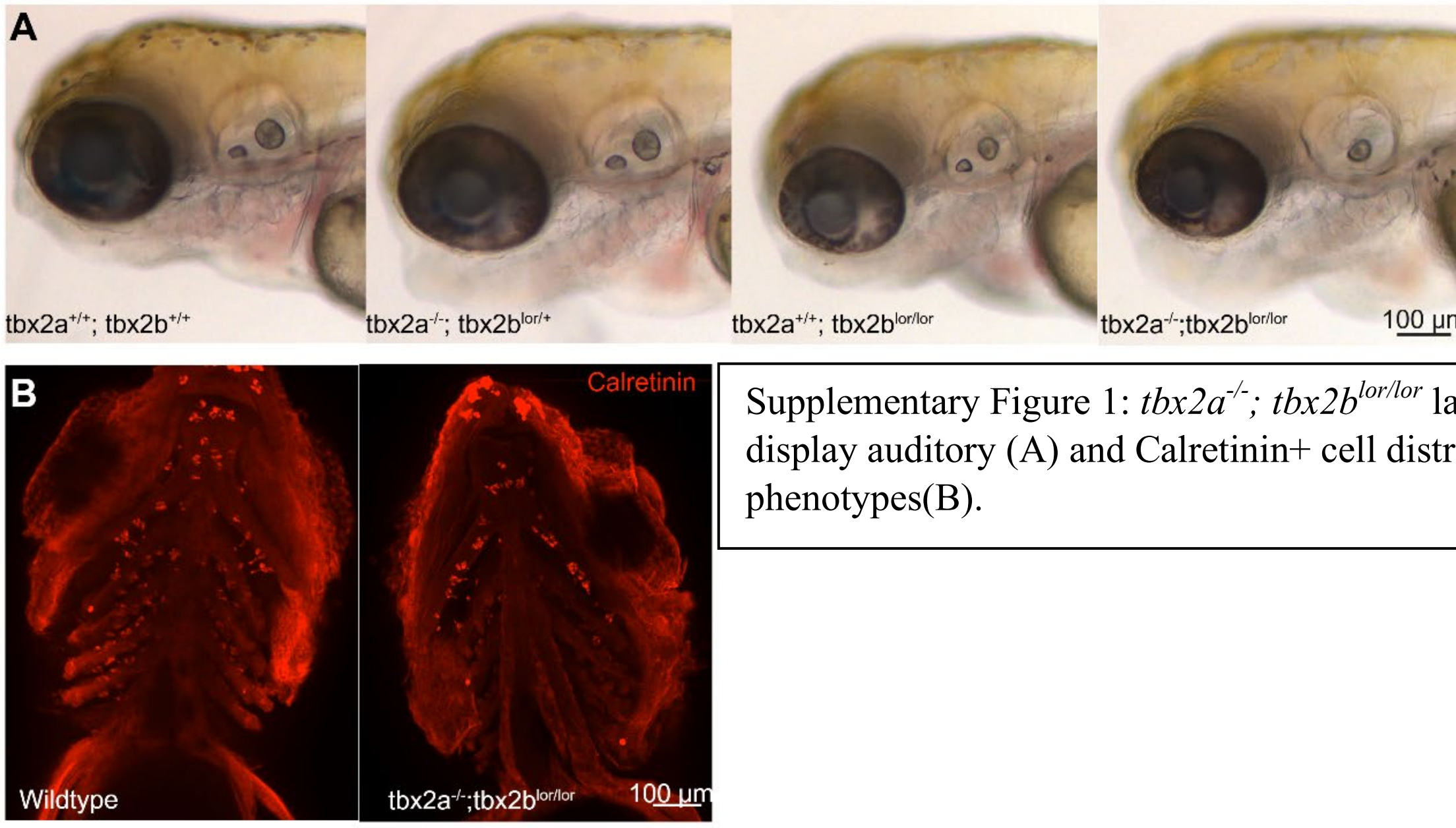

Supplementary Figure 1: *tbx2a*$^{-/-}$*; tbx2b*$^{lor/lor}$ larvae display auditory (A) and Calretinin+ cell distribution phenotypes(B).
